# Promotion of seed germination and early plant growth by KNO_3_ and light spectra in *Ocimum tenuiflorum* using a plant factory

**DOI:** 10.1038/s41598-022-11001-5

**Published:** 2022-04-29

**Authors:** Akira Thongtip, Kriengkrai Mosaleeyanon, Siripar Korinsak, Theerayut Toojinda, Clive Terence Darwell, Preuk Chutimanukul, Panita Chutimanukul

**Affiliations:** 1 National Center for Genetic Engineering and Biotechnology (BIOTEC), National Science and Technology Development Agency, Khlong Luang, 12120 Pathum Thani Thailand; 2 Department of Agricultural Technology, Faculty of Science and Technology, Thammasat University, Rangsit Centre, Khlong Nueng, Khlong Luang, 12120 Pathum Thani Thailand

**Keywords:** Plant biotechnology, Plant physiology

## Abstract

The plant factory with artificial light (PFAL) is a novel cultivation system of agriculture technology for crop production under controlled-environment conditions. However, there are a number of issues relating to low quality of seed germination and seedling vigor that lead to decreased crop yields. The present study investigates the optimal KNO_3_ concentration for seed germination, and the influence of different light spectra on early plant growth in holy basil (*Ocimum tenuiflorum*) under a PFAL system. Experiment 1 investigated the effects of KNO_3_ concentration (0, 0.2, 0.4 and 0.6%) on germination of seeds primed for 24 h under white Light emitting diodes (LED). Results show that sowing holy basil seeds in 0.4% KNO_3_ enhanced seed germination percentage (GP) and germination index (GI), while decreasing mean germination time (MGT). Experiment 2 investigated the effect of four light spectra on seed germination and early plant growth by sowing with 0 and 0.4% KNO_3_ and germinating for 15 days continuously under different monochromatic light settings: white, red, green and blue in PFAL. It was found that the green spectrum positively affected shoot and root length, and also decreased shortened MGT at 0 and 0.4% KNO_3_ when compared with other light treatments. Additionally, pre-cultivated seedlings under the green spectrum showed significant improvement in the early plant growth for all holy basil varieties at 15 days after transplanting by promoting stem length, stem diameter, plant width, fresh weights of shoot and root, and dry weights of shoot and root. These findings could be useful in developing seed priming and light treatments to enhance seed germination and seedling quality of holy basil resulting in increased crop production under PFAL.

## Introduction

Holy basil (*Ocimum tenuiflorum* L., Labiatae) is a self-pollinating plant found throughout tropical regions^1^. Holy basil can be divided into two major types using distinct morphological characters: (1) red or purple holy basil has dark green leaves with reddish purple stems, and (2) green or white holy basil has medium green leaves with light green stem^2^. It is also a well-known flavoring agent and essential oil that has antioxidative and antibacterial properties^3^. Holy basil is an aromatic herb commonly used to supplement a distinctive scent and taste. The leaves can be used fresh or dried for spice. Essential oils extracted from fresh leaves and flowers can be used as additives in food, pharmaceuticals and cosmetics^4^. Moreover, holy basil has traditionally been used treat a variety of diseases including headaches, coughs, respiratory illnesses, diarrhea, constipation and kidney malfunction^5^. Antioxidant activity of components in holy basil is one of main causes of its pharmacological actions. Phenolic compounds in holy basil extracts, including eugenol, cirsilineol, isothymusin, isothymonin, rosmarinic acid^6^, orientin, and vicenin^7^, have been identified as good antioxidant compounds, while zinc, an antioxidant mineral, has been found to be significantly high in holy basil^8^. The amount of these components varies on the kind of soil, as well as harvesting, processing, and storage methods^9^. These pharmacological findings have provided a scientific foundation for using holy basil in medicinal purposes. The great popularity of holy basil sets a continuing demand for seeds and thus for seed production^10^. Currently, holy basil is highly demanded in the food, cosmetic and pharmaceutical industries around the world, especially in South-east Asia countries, it is used to alleviate nausea, vomiting, antistress, anti-inflammatory, antidiabetic as well as reduce fevers, for coughs and colds, and for influenza^11–14^. Holy basil is an important commercial species with estimated annual trade of 2,000 to 5,000 metric tons in India^15^. In 2020, holy basil was used to as an adaptogens protect the body from physical and mental stress which is estimated to expand not less than 1.5 billion USD according to transparencymarketresearch.com. However, the agricultural industry faces a number of issues related to the low quality of seed germination and seedling vigor in holy basil, which leads to decrease in crop yields. Low seed germination has been shown to relate to the mucilaginous layer (seed gum)^16^. Abraham^16^ reported that the mucilage is apectinous matrix, consisting of considerable amounts of unesterified galacturonic acid with a large capacity for hydration that act as a reservoir blocking the absorption of oxygen while the seeds absorb water^17–19^. Moreover, the high accumulation in some phenolic and polyphenol compounds (benzoic acid and *p*-hydroxybenzoic acid) might exert potentially negative effects on seed germination and seedling development by decreasing enzyme activity in cellular functions and impairing the progression of cell division^20,21^. There are many ways to stimulate the germination of mucus-covered seeds, such as washing seed gum with ascorbic boric and HgCl_2_^22,23^ or soaking the seeds in a solution that promotes germination, such as GA_3_ KNO_3_ kinetin and ethrel^24^. Seed with mucous or hard seed coats can be improved by acid soaking before planting^20^. However, this method causes high seed mortality when used with large quantities of seeds^25^.

Seed priming involves preparing seeds before planting by soaking in water or chemical solution at the appropriate temperature and time-period. This is done to stimulate the early events of germination and various biochemical or metabolic processes, before drying the seed back to its original moisture condition^26^. It also causes reorganization of membrane and repairs damaged cells and organs^27^. Priming increase the uniformity, germination speed and the growth of seedling^28^. Seed priming includes hydropriming, hormonal priming, osmopriming, matrix priming. Hydropriming simply uses clean water and is therefore safe for the user, reduces costs, and does not leave toxic or chemical residues in either seeds nor the environment^29^. Osmopriming involves soaking of seeds in low water potential solutions and reduces the rate of water imbibition^27^. Osmotica used for seed priming include organic salts, such as polyethylene glycol (PEG), manitol and sorbitol, and are most commonly used to adjust osmotic potential. Inorganic salts, such as KNO_3_, CaCl_2_, KH_2_PO_4_^30^ increase nitrogen and other nutrients needed for protein synthesis while the seeds germinate. KNO_3_ is most widely used in seed priming to improve seed germination due to it is an ionic salt of potassium ions (K^+^) and nitrate ions (NO_3_^−^). It occurs as an alkali metal nitrate and is a major essential plant nutrient. Nitrogen is a component of many biomolecules in plant cells and helps seeds synthesize proteins, which has an impact on seed quality^31^. Additionally, K^+^ dissolves in the cytoplasm and vacuole and acts to maintain the osmotic potential. K^+^ is also associated in the stimulation of over 40 type of enzyme, especially enzymes in photosynthesis and respiration, as well as those used in the synthesis of starch and protein which help maintain the firmness of plant cells^32–35^. The duration of seed priming is critical and has been reported for many crop plants^28^. Seeds of pepper primed in PEG for six days produced more abnormal seedlings than seeds primed for four or five days^36^. In muskmelon studies, seeds are primed from 16 h^37^ to ten days^38^. Hydropriming at 12 h improve seedling vigor and germination percentage^39^, in addition it enhances seedling shoot and stem vigor index^40^ of basil.

One of the most important abiotic parameters for plant growth throughout the life cycle is light availability^41^. Solar light consists of electromagnetic energy with wavelengths ranging from 400 to 700 nm (violet, blue, green, yellow, orange and red). Plants have evolved light absorbing molecules that enable organisms to respond to changes in the light quality that affects various physiological processes depending on the species, developmental stage, or studied organ^42^. Furthermore, light acts as an environmental signal controlling the plant photomorphogenetic responses, including the transition from one development stage to the next^43^. Green light stimulates seed germination via the early elongation of the stems by antagonizing growth inhibition processes in *Arabidopsis*^44^. Blue light has a significant impact on seed germination by decreasing the germination percentage and mean germination time of *Brassica napus*^45^. In recent years, the utilization of plant factories with artificial lighting (PFAL) has become more commonplace for plant production purposes, and is considered an alternative crop plant production method in response to climate change^46,47^. PFAL is a modern agricultural system using advanced technologies for plant cultivation in a fully controlled environment, including: light, humidity, carbon dioxide, temperature, water and nutrient^48^. Light is one of the most important environmental factors affecting the plant growth, plant development and crop yields, especially in plant factory or indoor farming settings. In order to maximize the biomass productivity in a PFAL, it is critical to control the optimal light quantity (intensity and duration) and quality (wavelength composition) to improve the quantity and quality of crop plants. Currently, much research on the utilization of PFAL has been conducted on controlling seedlings in terms of stem diameter, hypocotyl length, fresh and dry weight, compactness and root development. Each is important to improve the success rate of plant production by manipulating the growth conditions^49–51^.

Moreover, holy basil is commercially cultivated through transplanting of seedlings, and often the farmer and commercial growers suffer from major losses due to low quality seeds. It is therefore essential to assess the germination potential and vigor in order to ensure an optimum crop stand and herb yield. Methods for improving sweet basil seed quality has been reported in various studies, however, there is no method to test for germination of holy basil^52^. Accordingly, this study aims to evaluate the optimal concentration of KNO_3_ for seed germination, and the influence of different monochromatic light spectra on seedling vigor and early plant growth in holy basil under a PFAL system. This study represents the first study reporting a priming effect of KNO_3_ concentration on seed germination and the influence of different monochromatic light on early plant growth of holy basil. Moreover, it contributes towards the improvement of seed technology utilizing LED technology.

## Materials and methods

### Seed source

Four varieties of holy basil, including two types of green and two types of red, were obtained from three commercial seed companies in Thailand as shown in Table 1.

**Table 1:** List of four holy basil varieties from three seed companies, brand, and coding name of holy basil plants grown in PFAL system.

Type	Company	Brand	Code
Green holy basil (G)	Choke Kasikorn seed Co., Ltd	Nam Tao	G-NT
	Tong Sam Co., Ltd	Tong Sam	G-TS
Red holy basil (R)	Chia Tai Co., Ltd	Kuang Bin	R-JT
	Tong Sam Co., Ltd	Tong Sam	R-TS

### Experiment 1: KNO_3_ concentration selection

#### Seed priming treatment

Seeds were randomly drawn from each variety and were primed with KNO_3_ solution at four different concentrations: 0%, (KNO3 0 g L^−1^) 0.2% (KNO_3_ 2.0 g L^–1^), 0.4% (KNO_3_ 4.0 g L^–1^) and 0.6% (KNO_3_ 6.0 g L^–1^) for 24 h at 25 ± 1 °C. The seeds were placed on Petri dishes with KNO_3_ solution, with petri dish lids then placed on top. Afterwards, seeds were rinsed thoroughly with reverse osmosis (RO) water and dried back to original moisture contents at room temperature for 2 days. Primed seeds with 0% KNO_3_ (hydropriming) were maintained as control for comparison.

#### Experimental site and germination test

After priming, seed germination tests were conducted. Four replicates of 50 seeds each were used for each treatment, placed on top of the filter paper with 10 mL of RO water in a Petri dish. Seeds of each treatment were set in each of the five shelves available in the PFAL. Seed germination tests were conducted in an environmentally-controlled room under PFAL system of National Center for Genetic Engineering and Biotechnology (BIOTEC), National Science and Technology Development Agency (NSTDA), Pathum Thani, Thailand. The environmental conditions consisted of 150 µmol m^−2^ s^−1^ of white LED lights for PPFD with 12 h d^−1^ photoperiod and 25 ± 1 °C temperature. Germinated seeds featuring cotyledons roots were counted every three days for 15 days. Then, the germination percentage (GP) on day 15 after sowing was calculated. The germination index (GI) and mean germination time (MGT) were calculated as GI = ∑(G_t_/T_t_) and MGT = ∑(G_t_ × T_t_)/∑G_t_, respectively^53,54^, where G_t_ is the number of germinated seeds on Day t, T_t_ is time corresponding to G_t_ in days. The results of KNO_3_ concentration from this experiment were taken into consideration for selecting the appropriate treatment to be used in the second experiment.

#### Statistical analysis

The experiment was arranged as a completely randomized design (CRD) with four replications and 50 seeds per replicate for each concentration. The priming concentration were 0%, 0.2%, 0.4% and 0.6% KNO_3_. GP, MGT and GI were subjected to analysis of variance (ANOVA). The differences between the means were compared using Duncan Multiple Range’s test (P < 0.05).

### Experiment 2: light spectrum on seed germination and seedling characteristics

#### Monochromatic light treatment and Statistical analysis

**Figure 1:**
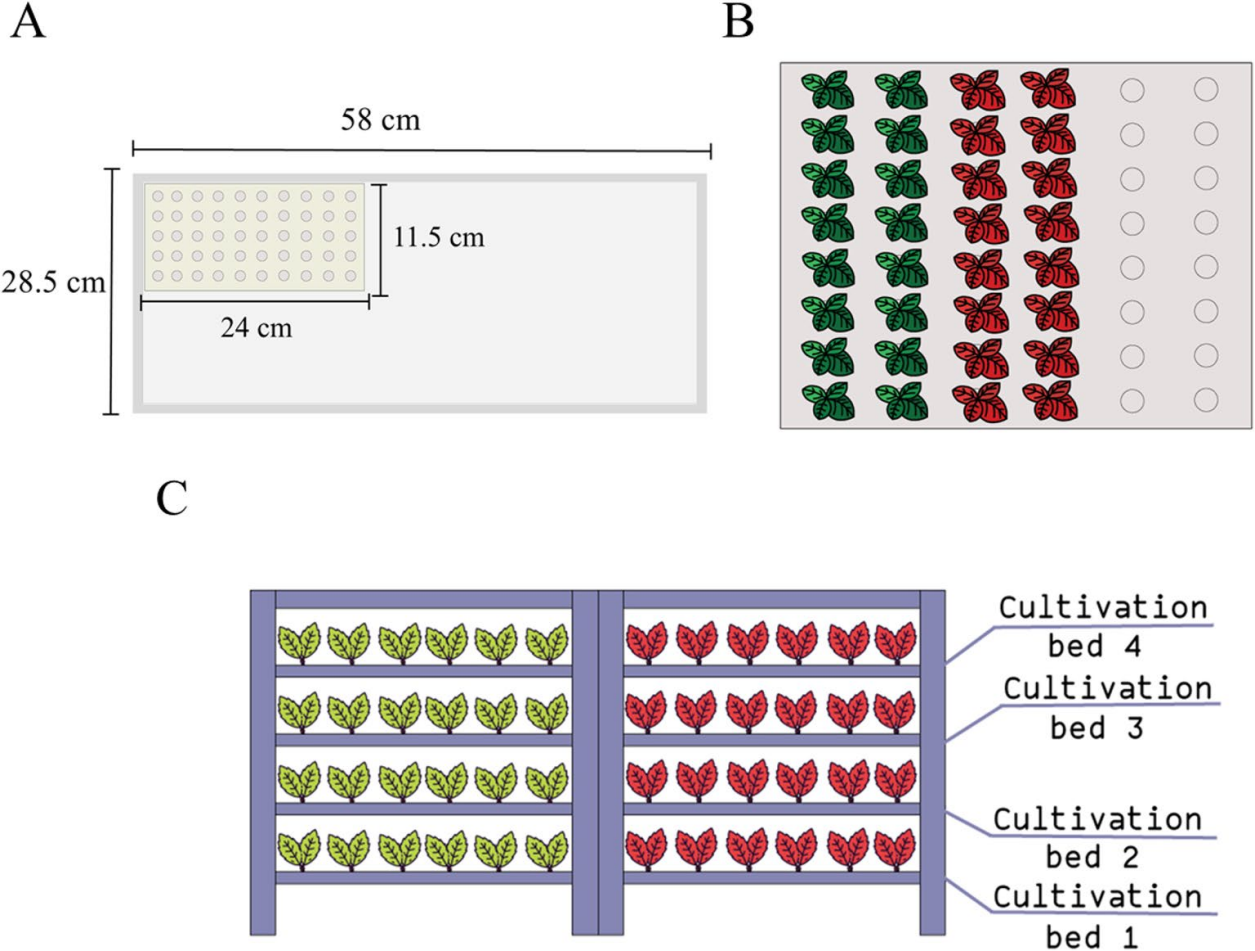
Top view of the sponge 50-cell germination (**A**), top view hydroponic foam board for early plant growth of holy basil (**B**) and side view of cultivation bed (4 cultivation beds) under fully control environment in the PFAL system.

According to the data form the first experiment, 0.4% KNO_3_ was chosen for seed priming in the PFAL system. Holy basil seeds were primed with 0% and 0.4% KNO_3_ for 24 h at 25 ± 1 °C. The seeds were rinsed with RO water and dried back to their original moisture contents (less than 15% moisture content) at room temperature for 2 days. Four biological replicates were performed for each sampling concentration, each one containing 50 seeds. The seeds from each variety were sown on a sponge mat with a 50-cell germination (cell size 24 × 11.5 × 3 cm) (ESPEC Corp., Japan), and placed on foam trays (Fig. 1A) containing RO water with water saturation content at 100% moisture content. For seed germination, a total 200 seeds of each variety from the trays were moved to the shelves under controlled environmental conditions. To maintain a high moisture content, the germination trays were covered with clear plastic sheets and watered daily with RO water. The environmental conditions consisted of 150 µmol m^−2^ s^−1^ of white LEDs, monochromatic blue lights (λ = 400–500 nm, peak at 450 nm), green (λ = 500–600 nm, peak at 525 nm) and red (λ = 600–700 nm, peak at 660 nm) (Supplementary Fig. S1) of PPFD with 12 h d^−1^ photoperiod, 70 ± 5% relative humidity (RH), 1000 ± 300 µmol mol^−1^ (ppm) CO_2_ concentration and 25 ± 1 °C temperature.

The number of germinated seeds was recorded daily over a period of 15 days. A seed was considered as germinated when the hypocotyl and cotyledons protruded from the seed coat. At the end of this period, the GP (%), MGT and GI were calculated. Root and shoot lengths were determined by digital photographs using ImageJ v 1.5.3e software^55^. The same randomly selected seedlings were collected and transferred to the hydroponics system to evaluate the seedling performance attributes after pre-treatment with different monochromatic light for 15 days.

The experiment was arranged as a completely randomized design (CRD) with 8 treatments (0% and 0.4% KNO_3_ combined with LEDs light that is white, red, green and blue light) each with four replications and each replicate of 50 seeds. Germination percentage, mean germination time and germination index were subjected to analysis of variance (ANOVA). The differences between the means were compared using Duncan Multiple Range’s test (P < 0.05).

#### Seedling performance and statistical analysis

Based on the monochromatic light treatments, holy basil seedlings at 15 days after sowing were transplanted into plant production room in the PFAL system which 200 µmol m^2^ s^−1^ white light for PPFD with 12 h d^−1^ photoperiod, 70 ± 5% relative humidity (RH), 1000 ± 300 µmol mol^−1^ (ppm) CO_2_ concentration and 25 ± 1 °C temperature. Seedlings featuring expanded cotyledons, elongated and hypocotyl and also enhanced root length were transferred to hydroponic foam boards (Fig 1B). Foam boards with seedlings were put into the cultivation bed (Fig 1C) and allowed to grow in a hydroponics system utilizing DFT (deep flow water technique). All seedlings in each treatment were grown in a dedicated compartment at the same electrical conductivity (EC) of the nutrient solution (0.8 mS cm^−1^). The selected plants were provided with a modified Enshi solution consisting of 220 g L^−1^ Ca(NO_3_)_2_·4H_2_O, 120 g L^−1^ KNO3, 120 g L^−1^ MgSO_4_·7H_2_O, 60 g L^−1^ KH2PO4, 10 g L^−1^ Fe-EDDHA, 10 g L^−1^ Fe-DTPA, 10 g L^−1^ NIC-SPRAY, 2 g L^−1^ Mn-EDTA, 100 mg L^−1^ NiSO_4_ 6H_2_O, 100 mg L^−1^ NaMoO_4_·H_2_O.

After 15 days of transplanting, plant growth parameters; stem length (cm), plant width (cm) and stem diameter (mm) were measured with a ruler and digital caliper. Shoot and root fresh weights were collected. Then, dry weight was obtained by drying in an oven set at 50 °C for 3 days, followed by weighing to determine average seedling dry weight per replication.

The experiment was arranged as a completely randomized design (CRD) with four replications and fifteen seedlings per replicate for each treatment. Four LED treatments primed with 0% and 0.4% KNO_3_ were subjected to analysis of variance (ANOVA). The differences between the means were compared using Duncan Multiple Range’s test (P < 0.05).

### Research involving plants

All the relevant institutional, national, and international guidelines and legislation have been followed.

## Results

### KNO_3_ concentration selection

**Figure 2:**
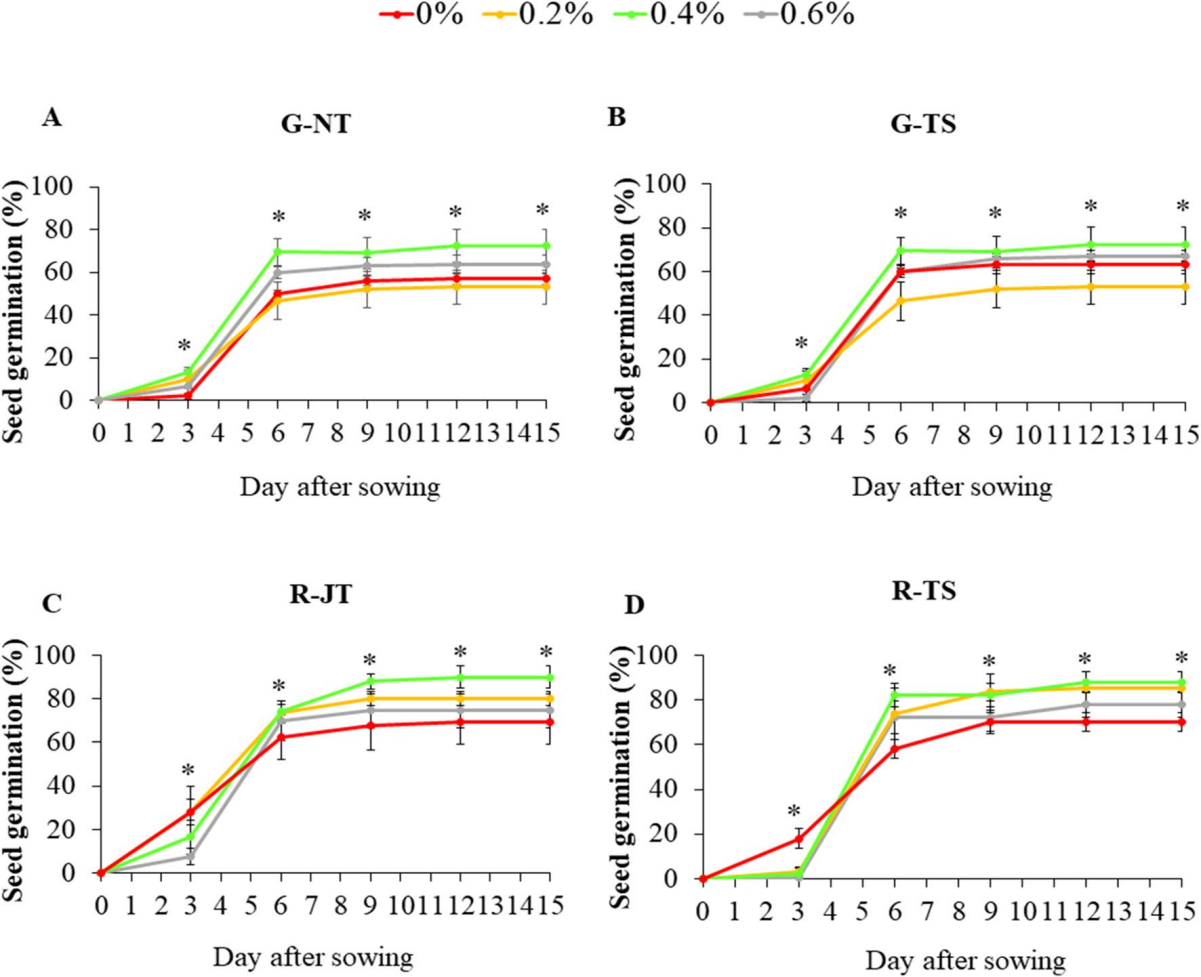
Seed germination percentage (%) after sowing in 0%, 0.2%, 0.4% and 0.6% of KNO_3_ concentration of four holy basil varieties from three companies; G-NT (**A**), G-TS (**B**), R-JT (**C**) and R-TS (**D**). Bars represent standard error of four biological replicates. The measurement was performed with fifty seeds in a replication. ANOVA was performed followed by mean comparison with DMRT.

Germination tests for four holy basil varieties including germination percentage (GP), mean germination time (MGT) and germination index (GI) of the seeds treated with different concentrations of KNO_3_ were calculated, and the results are shown in Table 2. The results of the current study indicate that seed priming with KNO_3_ improved the seed establishment of holy basil on the top of paper under 150 µmol m^−2^ s^−1^ of white LEDs at 25 °C. Holy basil seeds primed with 0.4% KNO_3_ had maximum GP and GI among three varieties, including G-NT (72.5%), R-JT (90%) and R-TS (88.0%). For MGT, there was no significant differences between the four concentrations of KNO_3_. Moreover, the patterns of seed germination of holy basil varieties for 15 days after sowing indicated the stimulatory impact of KNO_3_ (Fig. 2). The GP at 0.4% KNO_3_ showed a similar increasing pattern after six days of sowing among all four holy basil varieties. Thus, we focused our investigation on the effects of 0.4% KNO_3_ with monochromatic LEDs under fully controlled environment in plant factory system.

**Table 2:** Final seed germination percentage (GP) and mean emergence time (MGT) and germination index (GI) at 15 days after sowing of four holy basils as affected by seed priming with KNO_3_, on top of the filter paper with white LED.

KNO_3_ concentration	Seed germination (%)			
	G-NT	G-TS	R-JT	R-TS
0%	57.0 ± 1.29b	61.5 ± 6.45	74.5 ± 3.77b	78.0 ± 2.94bc
0.2%	53.0 ± 3.87b	59.5 ± 3.95	80.0 ± 1.63ab	85.5 ± 3.50ab
0.4%	72.5 ± 3.86a	57.5 ± 2.99	90.0 ± 2.58a	88.0 ± 2.45a
0.6%	63.5 ± 2.22a	48.5 ± 4.43	69.5 ± 5.12b	70.0 ± 2.16c
Significance	*	ns	*	*

KNO_3_ concentration	Mean germination time (day)			
	G-NT	G-TS	R-JT	R-TS
0%	6.2 ± 0.10	6.7 ± 0.31	6.0 ± 0.09	6.3 ± 0.13
0.2%	6.3 ± 0.26	6.5 ± 0.27	6.2 ± 0.16	6.3 ± 0.17
0.4%	6.1 ± 0.26	6.5 ± 0.11	6.6 ± 0.24	6.3 ± 0.13
0.6%	6.1 ± 0.13	6.3 ± 0.28	6.4 ± 0.12	6.5 ± 0.21
Significance	ns	ns	ns	ns

KNO_3_ concentration	Germination index			
	G-NT	G-TS	R-JT	R-TS
0%	5.0 ± 0.4bc	3.9 ± 0.30	5.8 ± 0.37a	9.3 ± 0.04ab
0.2%	4.2 ± 0.48c	4.1 ± 0.59	4.5 ± 0.45b	7.8 ± 0.57b
0.4%	6.8 ± 0.62a	3.3 ± 0.83	6.9 ± 0.56a	10.0 ± 0.93a
0.6%	5.2 ± 0.31bc	3.2 ± 0.30	4.3 ± 0.25b	4.4 ± 0.13c
Significance	*	ns	*	*

Data are mean values ± SE (n = 4) with four fifty seeds in a replication. Different letters in the same column indicate significant difference at p < 0.05. * indicates significant difference. “ns” indicates no significant difference.

### Seedling establishment with monochromatic LEDs

**Figure 3:**
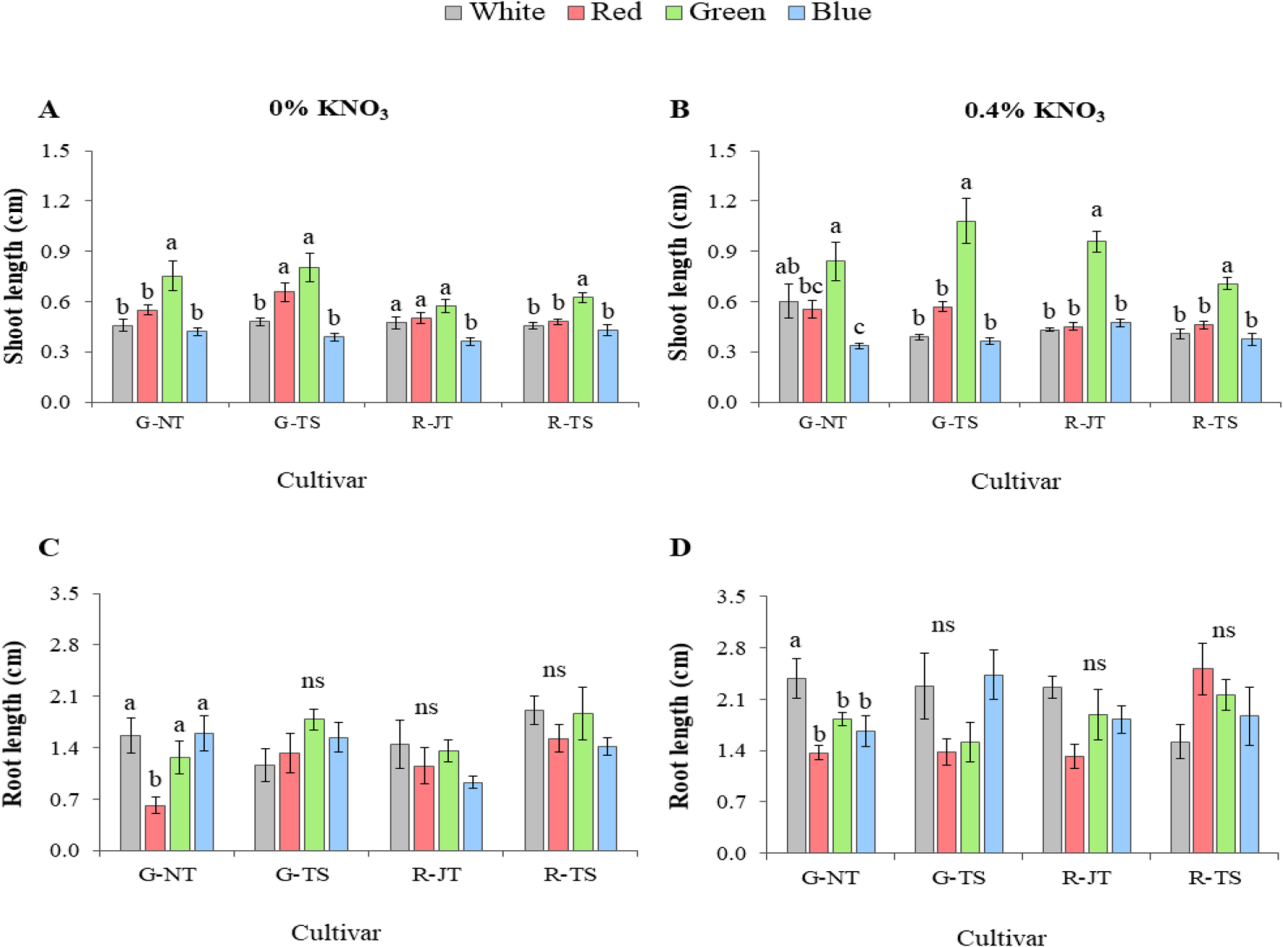
Shoot height (**A**, **B**) and root length (**C**, **D**) of four holy basil varieties from three companies; G-NT, G-TS, R-JT and R-TS treated with difference light treatments under 0% and 0.4% KNO_3_ concentrations at 15 days after sowing. Different letters above bars indicate significant differences among light treatments on the same brand at P < 0.05. “ns” indicates no significant difference. Data are mean values (n = 4) ± SE.

In this experiment, two concentrations of KNO_3_ at 0% (i.e. as control) and 0.4% were used for priming holy basil seeds for 24 h which were treated with four monochromatic LEDs under fully controlled conditions. Seedling establishment including GP, MGT and GI at 15 days after sowing (before transplanting) is shown in Table 3, the GP of the four holy basil varieties primed with 0% and 0.4% of KNO_3_ were not significantly different among light treatments (except R-JT at 0.4% KNO_3_). There were no significant differences observed in the GI parameter of the four light treatments across all varieties. Seeds primed with 0% and 4% KNO_3_ under monochromatic blue light (λ = 450 nm) and monochromatic green light (λ = 523 nm) resulted in a lower MGT than white LEDs and monochromatic red light (λ = 660 nm), while maximum MGT was observed with white LEDs. Although all seed varieties treated with monochromatic blue light germinated earlier, shoot lengths were shorter than for green light. Only monochromatic green light showed significant improvement in shoot length for all four varieties in both priming treatments at 0% and 0.4% KNO_3_ at 15 days after sowing (Fig. 3A,B), However, light treatments did not exert any significant effect on root length (Fig. 3C,D). The phenotypes of holy basil seedlings under four light treatments at 15 days after sowing (before transplanting in PFAL system) are shown in Supplementary Fig. S2. The results indicate that monochromatic green light can decrease germination time and promote shoot length growth before transplanting among all four holy basil varieties.

**Table 3:** Final seed germination percentage (GP) and mean emergence time (MGT) and germination index (GI) before transplanting at 15 days after sowing of four holy basil varieties by seed priming with KNO_3_, under different LED light treatments; white (W), monochromic red (R), green (G) and blue (B) in the PFAL system.

KNO_3_ concentration	Light treatment	Seed germination (%)			
		G-NT	G-TS	R-JT	R-TS
0%	W	55.5 ± 7.50	63.0 ± 4.93	47.0 ± 4.80	81.5 ± 1.71
	R	73.5 ± 2.36	65.5 ± 3.77	65.0 ± 7.37	80.0 ± 1.15
	G	69.0 ± 2.89	57.0 ± 4.20	57.5 ± 6.70	77.0 ± 3.42
	B	61.5 ± 5.12	52.0 ± 3.16	52.5 ± 4.99	79.0 ± 3.70
Significance		ns	ns	ns	ns
0.4%	W	71.5 ± 4.92	56.5 ± 2.87	55.5 ± 3.20ab	76.5 ± 5.12
	R	62.5 ± 3.59	58.5 ± 2.06	51.5 ± 5.19bc	82.0 ± 2.16
	G	59.5 ± 9.32	63.0 ± 5.45	67.5 ± 3.86a	78.0 ± 2.71
	B	62.5 ± 4.50	60.0 ± .2.94	40.5 ± 4.35c	81.0 ± 2.08
Significance		ns	ns	*	ns

KNO_3_ concentration	Light treatment	Mean germination time (day)			
		G-NT	G-TS	R-JT	R-TS
0%	W	6.0 ± 0.29a	6.1 ± 0.52a	6.1 ± 0.27a	6.18 ± 0.23a
	R	5.9 ± 0.07a	6.0 ± 0.13a	5.7 ± 0.18a	5.47 ± 0.18b
	G	5.2 ± 0.15b	4.7 ± 0.14b	4.4 ± 0.16b	5.21 ± 0.3b
	B	5.2 ± 0.21b	5.4 ± 0.26ab	5.7 ± 0.56a	5.23 ± 0.12b
Significance		*	*	*	*
0.4%	W	7.8 ± 0.78a	6.2 ± 0.61a	5.4 ± 0.15	5.87 ± 0.19a
	R	5.2 ± 0.20b	6.3 ± 0.22a	5.3 ± 0.24	5.18 ± 0.09ab
	G	4.6 ± 0.25b	4.9 ± 0.11b	5.2 ± 0.62	5.5 ± 0.62ab
	B	5.0 ± 0.19b	4.9 ± 0.32b	6.7 ± 0.53	4.7 ± 0.03b
Significance		*	*	ns	*

KNO_3_ concentration	Light treatment	Germination index			
		G-NT	G-TS	R-JT	R-TS
0%	W	20.5 ± 0.54	21.4 ± 0.99	20.7 ± 0.55	21.7 ± 0.45
	R	20.7 ± 0.19	19.9 ± 0.42	20.3 ± 0.60	22.2 ± 0.25
	G	21.8 ± 0.70	20.4 ± 0.50	20.8 ± 0.87	22.1 ± 0.44
	B	21.3 ± 0.44	20.8 ± 0.57	22.9 ± 0.89	23.0 ± 0.45
Significance		ns	ns	ns	ns
0.4%	W	23.8 ± 1.26	20.4 ± 0.36	20.7 ± 0.27	21.7 ± 0.74
	R	20.4 ± 0.40	19.5 ± 0.32	19.5 ± 0.32	23.0 ± 0.58
	G	21.0 ± 1.38	21.5 ± 0.68	21.9 ± 0.86	22.8 ± 1.06
	B	22.0 ± 0.58	21.6 ± 1.12	21.8 ± 0.86	23.8 ± 0.67
Significance		ns	ns	ns	ns

Data are mean values ± SE (n = 4) with four fifty seeds in a replication. Different letters in the same column indicate significant difference at p < 0.05. * indicates significant difference. “ns” indicates no significant difference.

### Early plant growth under PFAL system

**Figure 4:**
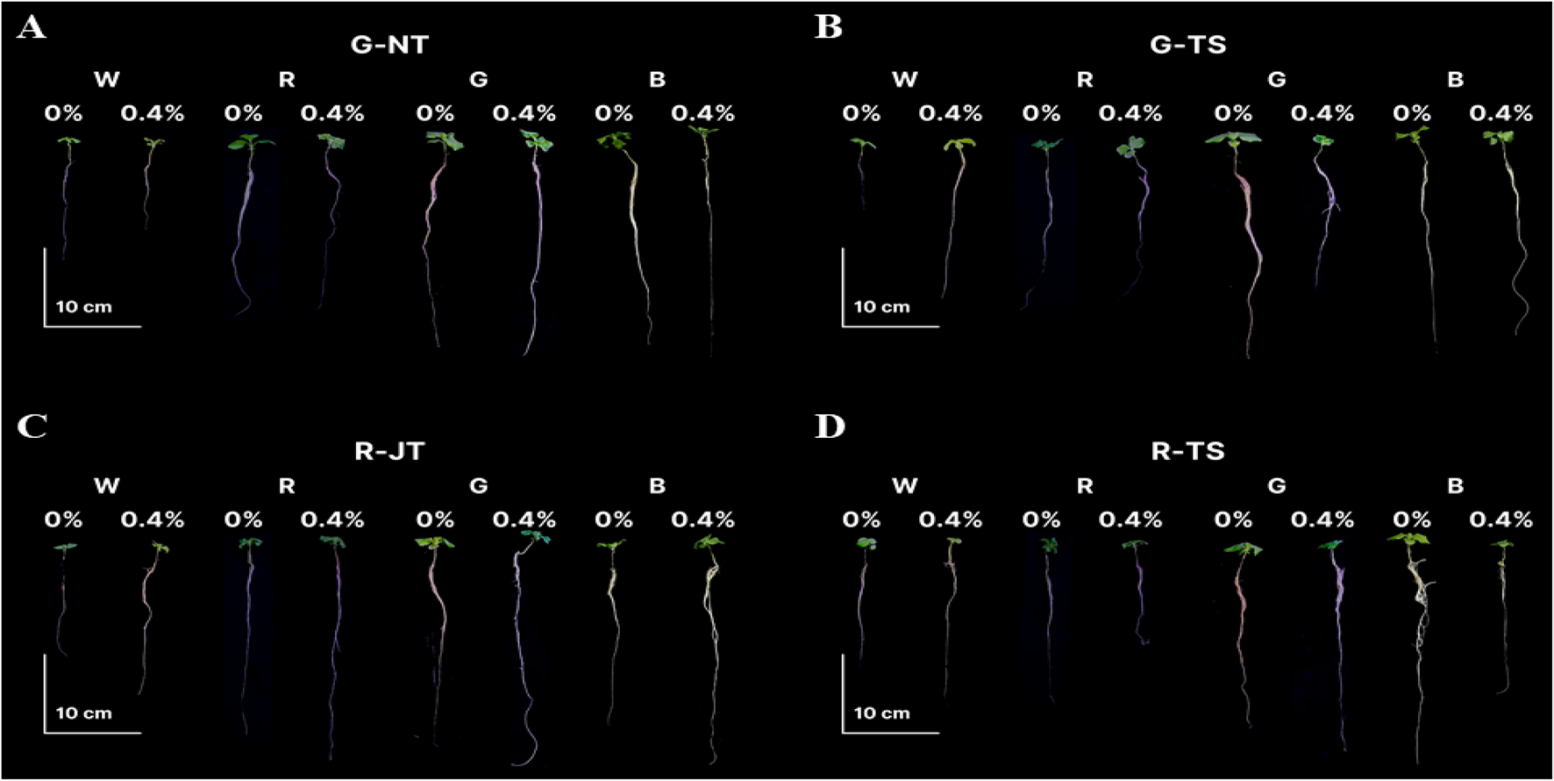
Early plant growth from four light treatments; white LEDs (W), monochromic red (R), green (G) and blue (B) LEDs with 0% and 4% of KNO3 priming among four holy basil varieties; G-NT (**A**), G-TS (**B**), R-JT (**C**) and R-TS (**D**) subsequently grown with white LEDs for 15 days under fully controlled environments.

**Figure 5:**
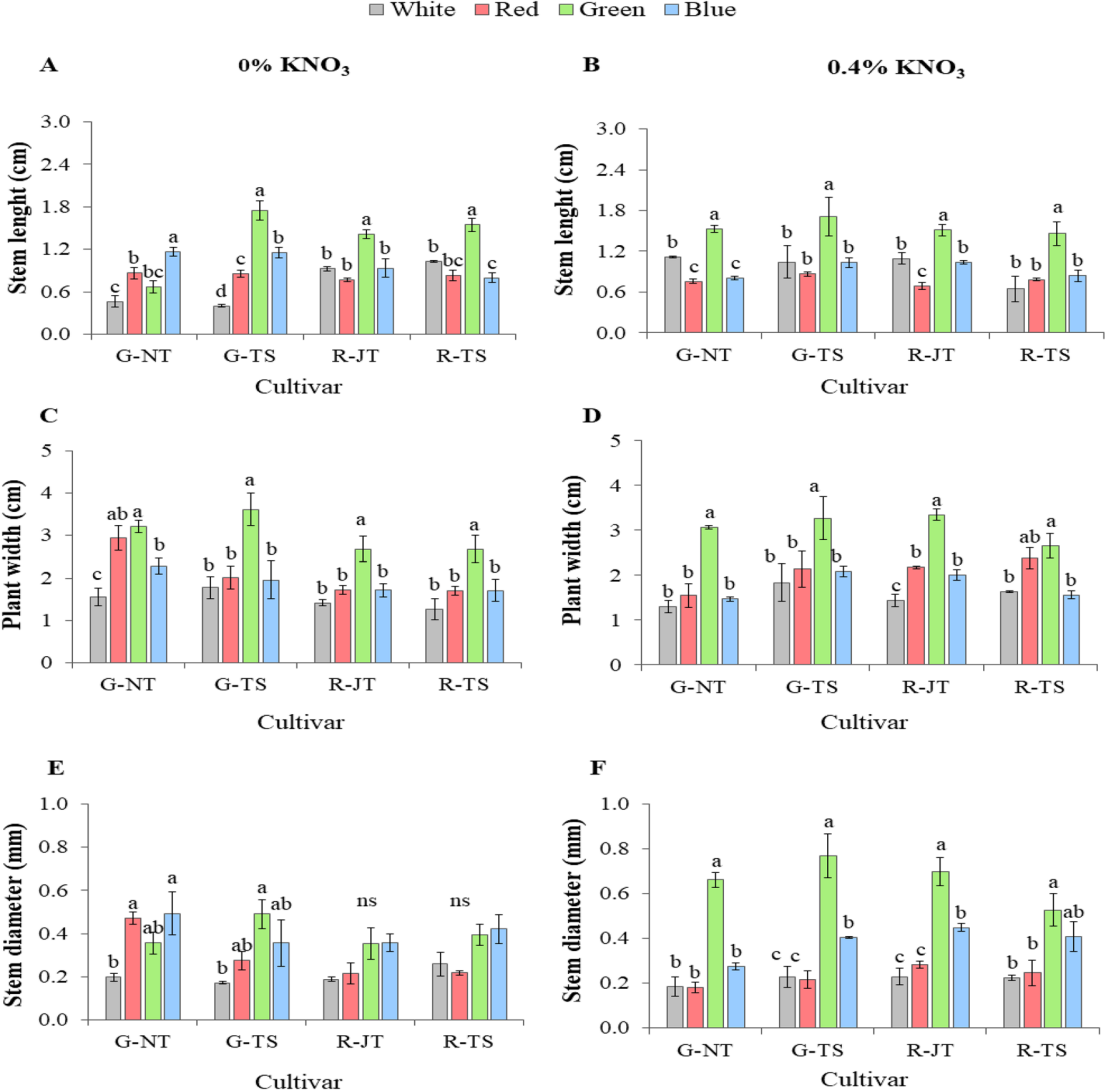
Stem length (**A**, **B**), plant width (**C**, **D**) and stem diameter (**E**, **F**) from four light treatments; white LEDs (W), monochromic red (R), green (G) and blue (B) LEDs with 0% and 4% of KNO3 priming among four holy basil varieties; G-NT (**A**), G-TS (**B**), R-JT (**C**) and R-TS (**D**) subsequently grown with white LEDs for 15 days under fully controlled environments. Different letters above bars indicate significant differences within group of the same basil at P < 0.05. Data are mean values (n = 4) ± SE.

**Figure 6:**
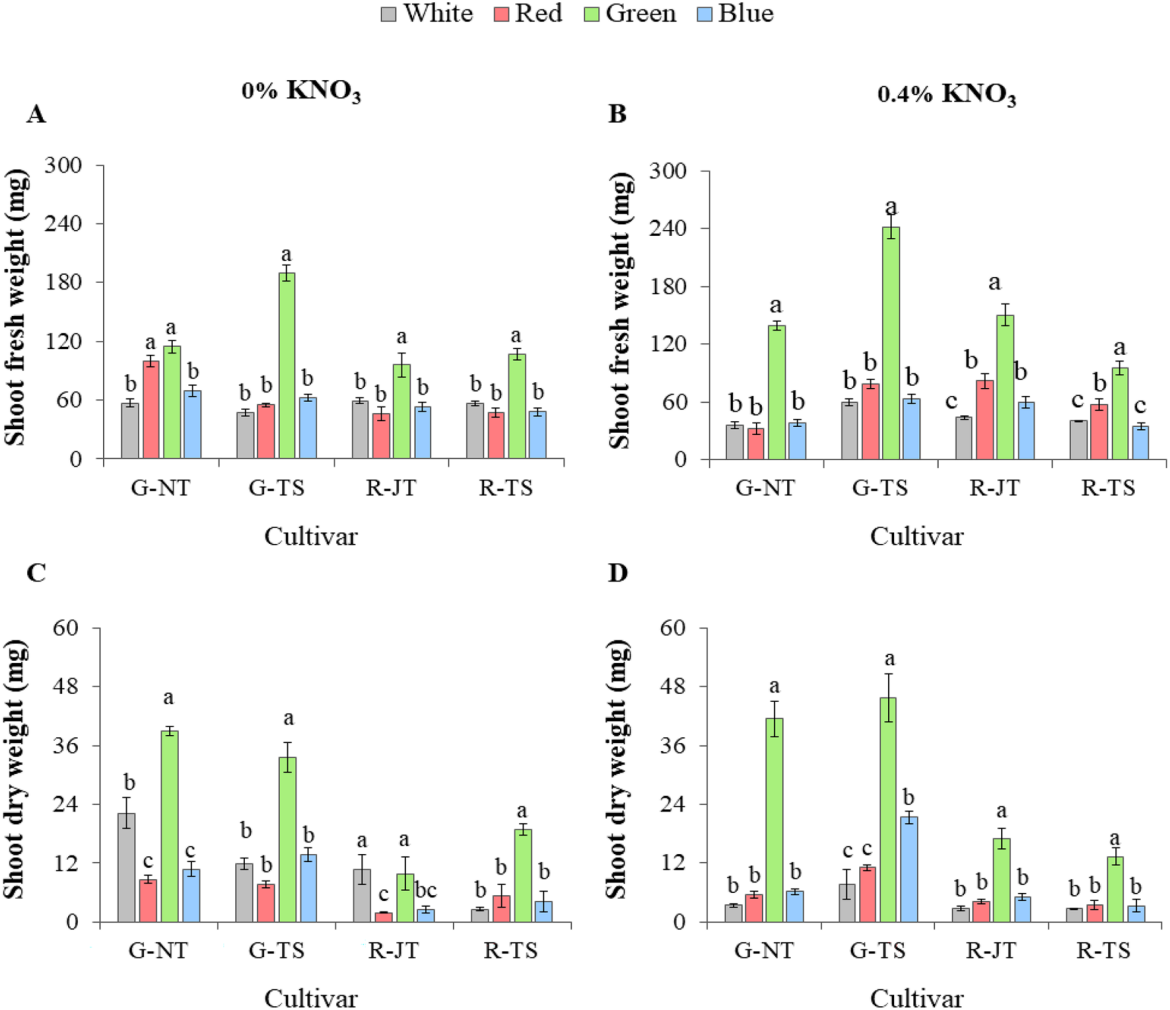
Fresh weight (**A**, **B**) and dry weight (**C**, **D**) of shoots from four light treatments; white LEDs (W), monochromic red (R), green (G) and blue (B) LEDs with 0% and 4% of KNO3 priming among four holy basil varieties; G-NT (**A**), G-TS (**B**), R-JT (**C**) and R-TS (**D**) subsequently grown with white LEDs for 15 days under fully controlled environments. Different letters above bars indicate significant differences within group of the same basil at P < 0.05. Data are mean values (n = 4) ± SE.

Based on the above results, all holy basil seedlings from each monochromatic light treatment with 0% and 0.4% KNO_3_ priming were transplanted under fully controlled conditions in the plant factory to determine the effects on early plant growth. Morphology of the plants 15 days after transplanting is displayed in Fig. 4. Morphological evaluation of seedling quality, including stem length, plant width and stem diameter of four varieties at 15 days after transplanting are presented in Fig. 4. Data revealed that seedlings under green light treatment primed with both KNO_3_ concentrations featured significantly greater stem length and plant width when compared to those from white, red and blue light treatments (Fig. 5A–D). Seedlings from three monochromic lights with 0% KNO_3_ priming showed no significant differences for the stem diameter in G-NT and G-TS, however, it was higher than white LEDs (Fig. 5E). Nonetheless, seedlings of all varieties under green light treatment with 0.4% KNO_3_ priming had a significant greater or broader stem diameter compared to light treatments (Fig. 5F). Green light treatment also showed greater fresh and dry weight accumulation of shoot after growing with white LEDs for 15 days (Fig. 6A,B). Seedlings with pre-treatment for 15 days with monochromatic green light also induced greater root fresh weight in all varieties when compared to other light treatments at 15 days after transplanting; however, there were significant differences in root dry weight among light treatments with 0.4% KNO_3_ priming, except for both red holy basils. (Supplementary Table S1).

In general, our study indicates that monochromic green light might be the best available method owing to higher complete germination viability and seedling establishment leading to plant vigor and plant development.

## Discussion

Seed priming improved both seedling growth and reduction of seedling germination time leading to higher grain yields^56,57^. Priming enhances the metabolic processes during early phase of germination before radicle protrusion and improves seed respiration processes^58,59^. Moreover, seed priming with KNO_3_ has been shown to influence final seed germination percentages by decreasing the germination times, seed vigor and uniformity of germination in various plant species^60–65^. Similarly in our study, we found the difference in germination performances between KNO_3_ concentrations. Results from experiment 1 showed that KNO_3_ priming influenced seed germination percentage, mean germination time and germination index among four holy basil varieties. Moreover, the results shown in Table 2 indicate that priming of holy basil seeds with 0.4% KNO_3_ caused greater final GP and GI than other concentrations. Several studies have found that higher concentrations of KNO_3_ for seed priming affect seed germination by inhibiting GP and MGT in many plants^65–68^. A similar trend in slight suppression of seed germination pattern in this study was observed for high concentrations of KNO_3_ (0.6%) across all holy basil varieties. The findings of the present study indicate that the performance of all holy basil varieties primed with 0.4% KNO_3_ was appreciably improved when subsequently germinated on top of the filter paper with white LEDs in plant factory. The pattern of seed germination and germination index were almost the same for the four varieties (Table 2), meaning that seed priming with 0.4% KNO_3_ was useful in terms of GP and GI for all holy basil varieties. In contrast, seed priming with 0% and 0.4% of KNO_3_ did not significantly influence GP and GI according to light treatments under fully controlled environment in PFAL using the sponge mat (Table 3). There are some reports explaining the performance of seed germination according to many factors including types of substrate, growth media, and also environmental factors such as light, oxygen, water, temperature and plant species^69,70^. A good substrate provides adequate high water retention capacity necessary for seed germination of holy basil. Since the water absorption rate and biochemical reactions are also key factors to stimulate the seed germination, the increase in seed germination percentage could be a reflection of the high moisture content in agricultural substrate^71,72^.

Recently, many research studies on the utilization of LED technology on plant growth, development, and morphology have been carried out under in vitro conditions and as indoor experiments^73,74^. Control of seedling growth and quality traits such as hypocotyl length, root length and stem diameter is very important in promoting success rate of plant production under manipulated environmental conditions^50^. However, the influence of light spectra on seed germination and early plant growth under PFAL systems has been rarely reported. In the present study, promotion of both seed germination and seedling quality under monochromatic lights is reported for the first time in holy basil. The results of this study demonstrate that monochromatic green light results in decreased MGT and significantly promotes shoot length when compared with other light treatments of holy basil primed with 0.4% KNO_3_ (at 15 days after sowing). The results are consistent across the four holy basil varieties and have the same trends for MGT and shoot length (Fig. 3 and Table 3). These results are also in agreement with observations of Pierik^75^ and Zhang^76^ who reported that seedlings grown under green light also commonly show typical shade avoidance responses such as increased shoot growth and plant height. Moreover, green light is efficiently absorbed in inner canopy levels during plant photosynthesis (Folta and Maruhnich^77^) and might stimulate growth through phytochrome and cryptochrome activity leading to morphogenetic change^78^. Sweet basil (*Ocimum basilicum* L.) plants were positively affected by green light when measuring plant biomass, plant dry matter, steam length, and number of leaves at 50 µmol m^–2^ s^−1^^79^. Moreover, concentration of volatile organic compounds were higher at 150–200 µmol m^−2^ s^−1^ of green light^80,81^.

Under early plant growth after 15 days of transplanting, the seedling from green light pre-treatment caused the greatest effects on stem length, plant width, and stem diameter in holy basil seedling (Fig. 5). This would likely greater more leaf expansion and shoot length, and thereby cause plants to accumulate increased total carbon gain and lead to faster growth and biomass^82^. Interestingly, our result showed the same increased tendency in fresh and dry weights of shoot for all holy basil varieties (Fig. 6). Thus, seed germination, and seedling vigor are the two essential trait parameters determining the success in crop production^83^. These results suggest that pre-treatment under monochromatic green light had a significant effect on seedling quality before transplanting as well as for early plant growth that may positively affect plant vegetative growth. In addition, the monochromatic green light triggered specific responses in the growth and morphology of holy basil seedlings that were demonstrable even after the seedling transplanting to PFAL conditions. Still, further research is needed to understand the influence of monochromatic light spectrum on pigments, secondary metabolites and antioxidant systems during seed germination, which may also contribute to greater understanding in enhancing the productivity of many other economically important crops.

## Conclusions

The results demonstrate an effective seed priming (with KNO_3_) and pre-treatment with light spectrum approach for promoting seed germination and seedling performance attributes among four holy basil varieties sourced from three seed companies. Seed priming performed at several concentrations of KNO_3_ found the highest seed germination rate at 0.4% of KNO_3_ while placed on top of the filter paper with white LEDs. Under plant production processing in PFAL conditions, GP and GI of all holy basils did not differ between monochromatic light treatments. However, green light spectrum showed high seedling improvement before transplanting by reducing MGT and increasing shoot length. Furthermore, results after transplanting confirm the advantage of using green light spectrum to increase the production of high quality seedlings in holy basil. This should be a successful approach for improving the speed of seed germination, seedling establishment and seedling vigor, thus enhancing the productivity of holy basil under PFAL system.

## Supplementary Information

Supplementary Information.

## References

[1] Nation RG, Janick J, Simon JE (1992). Estimation of outcrossing in basil. HortScience.

[2] Wangcharoen W, Morasuk W (2007). Antioxidant capacity and phenolic content of holy basil. Songklanakarin J. Sci. Technol..

[3] Javanmardi J, Stushnoff C, Locke E, Vivanco J (2003). Antioxidant activity and total phenolic content of Iranian Ocimum accessions. Food Chem..

[4] Javanmardi J, Khalighi A, Kashi A, Bais H, Vivanco J (2002). Chemical characterization of basil (*Ocimum basilicum* L.) found in local accessions and used in traditional medicines in Iran. J. Agric. Food Chem..

[5] Simon J, Morales M, Phippen W, Vieira R (1999). & Hao, Z.

[6] Kelm M, Nair M, Strasburg G, DeWitt D (2000). Antioxidant and cyclooxygenase inhibitory phenolic compounds from *Ocimum sanctum* Linn. Phytomedicine.

[7] Vrinda B, Devi PU (2001). Radiation protection of human lymphocyte chromosomes in vitro by orientin and vicenin. Mutat. Res..

[8] Samudralwar DL, Garg AN (1996). Minor and trace elemental determination in the Indian herbal and other medicinal preparations. Biol. Trace Elem. Res..

[9] Singh N, Verma P, Pandey B, Bhalla M (2012). Therapeutic potential of *Ocimum sanctum in* prevention and treatment of cancer and exposure to radiation: An overview. Int. J. Pharm. Sci. Drug Res..

[10] Putievsky E, Galambosi B (1999). Basil.

[11] Nepal I (2000). National Register of Medicina I Plants.

[12] Cohen MM (2014). Tulsi-*Ocimum sanctum*: A herb for all reasons. J. Ayurveda Integr. Med..

[13] Ansari A (2015). Study of plant tulsi and its benefits for human beings. Int. J. Appl. Res..

[14] Shasany AK (2016). The Holy basil (*Ocimum sanctum* L.) and its genome. Indian J. Hist. Sci.

[15] Ved D, Goraya G (2008). Demand and supply of medicinal plants. Medplant ENVIS Newslett. Med. Plants.

[16] Fahn A, Werker E (1972). Anatomical mechanisms of seed dispersal. Seed Biol. Import. Dev. Germin..

[17] Anjaneyalu YV, Khan M-R, Tharanathan RN (1983). An acidic xylan from the capsular polysaccharide-complex of *Ocimum gratissimum* seeds. Carbohyd. Res..

[18] Yang X, Dong M, Huang Z (2010). Role of mucilage in the germination of Artemisia sphaerocephala (Asteraceae) achenes exposed to osmotic stress and salinity. Plant Physiol. Biochem..

[19] Bewley JD, Black M (2012). Physiology and Biochemistry of Seeds in Relation to Germination: Volume 2: Viability, Dormancy, and Environmental Control.

[20] Dey, B. & Choudhuri, M. Seed germination as affected by plant age, growth and development stages of Ocimum sanctum. *Seed Sci. Technol.* (1982).

[21] Baleroni C, Ferrarese M, Souza N, Ferrarese-Filho O (2000). Lipid accumulation during canola seed germination in response to cinnamic acid derivatives. Biol. Plant..

[22] Mishra A, Choudhuri M (1998). Amelioration of lead and mercury effects on germination and rice seedling growth by antioxidants. Biol. Plant..

[23] Khafagy M, Arafa A, El-Banna M (2009). Glycinebetaine and ascorbic acid can alleviate the harmful effects of NaCl salinity in sweet pepper. Aust. J. Crop Sci..

[24] Debeaujon I, Lepiniec L, Pourcel L, Routaboul J-M (2007). Seed coat development and dormancy. Seed Dev. Dormancy Germin..

[25] Heydecker, W. & Coolbear, P. Seed treatments for improved performance survey and attempted prognosis. *Seed Sci. Technol.* (1977).

[26] Derek Bewley J (1986). Membrane changes in seeds as related to germination and the perturbations resulting from deterioration in storage. Physiol. Seed Deterior..

[27] McDonald M (1999). Seed deterioration: Physiology, repair and assessment. Seed Sci. Technol..

[28] Bradford KJ (1986). Manipulation of seed water relations via osmotic priming to improve germination under stress. HortScience.

[29] Huang R, Sukprakarn S, Phavaphutanon L, Juntakool S, Chaikul C (2006). Changes in antioxidant enzyme activity, lipid peroxidation and seedling growth of cucumber seed induced by hydropriming and electric field treatments. Agric. Nat. Resour..

[30] Frett J, Pill W, Morneau D (1991). A comparison of priming agents for tomato and asparagus seeds. HortScience.

[31] Vance CP (2001). Symbiotic nitrogen fixation and phosphorus acquisition: Plant nutrition in a world of declining renewable resources. Plant Physiol..

[32] Anschütz U, Becker D, Shabala S (2014). Going beyond nutrition: Regulation of potassium homoeostasis as a common denominator of plant adaptive responses to environment. J. Plant Physiol..

[33] Kafkafi U, Xu G, Imas P, Magen H, Tarchitzky J (2001). Potassium and Chloride in Crops and Soils: The Role of Potassium Chloride Fertilizer in Crop Nutrition.

[34] Wang Y, Wu W-H (2013). Potassium transport and signaling in higher plants. Annu. Rev. Plant Biol..

[35] Hirsch RE, Lewis BD, Spalding EP, Sussman MR (1998). A role for the AKT1 potassium channel in plant nutrition. Science.

[36] Cantliffe, D. *et al*. in *Proceedings of the... annual meeting of the Florida State Horticulture Society* (USA) (1988).

[37] Dhillon NS (1995). Seed priming of male sterile muskmelon (*Cucumis melo* L.) for low temperature germination. Seed Sci. Technol..

[38] Yeoung, Y., Wilson Jr, D. & Murray, G. Germination performance and loss of late-embryogenesis-abundant (LEA) proteins during muskmelon seed priming. *Seed Sci. Technol. *(1996).

[39] Farahani HA, Maroufi K (2011). Effect of hydropriming on seedling vigour in basil (*Ocimum basilicum* L.) under salinity conditions. Adv. Environ. Biol..

[40] Noorhosseini SA, Jokar NK, Damalas CA (2018). Improving seed germination and early growth of garden cress (*Lepidium sativum*) and basil (*Ocimum basilicum*) with hydro-priming. J. Plant Growth Regul..

[41] Fankhauser C, Chory J (1997). Light control of plant development. Annu. Rev. Cell Dev. Biol..

[42] He J, Qin L, Chong EL, Choong T-W, Lee SK (2017). Plant growth and photosynthetic characteristics of *Mesembryanthemum crystallinum* grown aeroponically under different blue-and red-LEDs. Front. Plant Sci..

[43] Devlin PF, Christie JM, Terry MJ (2007). Many hands make light work. J. Exp. Bot..

[44] Burescu L, Cachita D, Craciun C (2015). The effect of different wavelengths LED lighting on the growth of spruce (*Picea abies* L) plantlets. Roman. Biotechnol. Lett..

[45] Tehrani PF, Majd A, Mahmoodzadeh H, Satari TN (2016). Effect of red and blue light-emitting diodes on germination, morphological and anatomical features of *Brassica napus*. Adv. Stud. Biol.

[46] Kozai T, Niu G (2020). Plant Factory.

[47] Kozai T, Niu G, Takagaki M (2019). Plant Factory: An Indoor Vertical Farming System for Efficient Quality Food Production.

[48] Kozai T (2015). PFAL Business and R&D in the World: Current Status and Perspectives.

[49] An S (2021). Evaluation of air temperature, photoperiod and light intensity conditions to produce cucumber scions and rootstocks in a plant factory with artificial lighting. Horticulturae.

[50] An S, Park SW, Kwack Y (2020). Growth of cucumber scions, rootstocks, and grafted seedlings as affected by different irrigation regimes during cultivation of ‘Joenbaekdadagi’ and ‘Heukjong’ seedlings in a plant factory with artificial lighting. Agronomy.

[51] Chutimanukul P (2022). The influence of different light spectra on physiological responses, antioxidant capacity and chemical compositions in two holy basil cultivars. Sci. Rep..

[52] ISTA (2018). International rules for seed testing.

[53] Zhang S, Hu J, Zhang Y, Xie X, Knapp A (2007). Seed priming with brassinolide improves lucerne (*Medicago sativa* L.) seed germination and seedling growth in relation to physiological changes under salinity stress. Austral. J. Agric. Res..

[54] Kaya M (2008). Interaction between seed size and NaCl on germination and early seedling growth of some Turkish cultivars of chickpea (*Cicer arietinum* L.). J. Zhejiang Univ. Sci. B.

[55] Rasband, W. (2015).

[56] Abou-Zeid HM, Moustafa Y (2014). Physiological and Cytogenetic Responses of Wheat and Barley to Silver Nanopriming Treatment.

[57] Bhati-Kushwaha H, Kaur A, Malik C (2013). The synthesis and role of biogenic nanoparticles in overcoming chilling stress. Indian J. Plant Sci..

[58] Bray CM (2017). Seed Development and Germination.

[59] Singh G, Gill S, Sandhu KK (1999). Improved performance of muskmelon (*Cucumis melo*) seeds with osmoconditioning. Acta Agrobot..

[60] Bush EW, Wilson P, Shepard DP, McClure G (2000). Enhancement of seed germination in common carpetgrass and centipedegrass seed. HortScience.

[61] Madakadze R, Chirco EM, Khan AA (1993). Seed germination of three flower species following matriconditioning under various environments. J. Am. Soc. Hortic. Sci..

[62] McDonald M (2000). Seed Priming. Seed Technology and Its Biological Basis.

[63] Abdel-Baki GK, Shaddad M, Mostafa D, Rafat A-S (2018). The effect of seed presoaking with KNO3 on seed germination, proline, protein pattern, ß-amylase and mineral composition of two faba bean cultivars treated with NaCl. Egypt. J. Bot..

[64] Moaaz Ali M (2020). Effect of seed priming with potassium nitrate on the performance of tomato. Agriculture.

[65] Bian L, Yang L, Wang J-A, Shen H-L (2013). Effects of KNO 3 pretreatment and temperature on seed germination of *Sorbus pohuashanensis*. J. For. Res..

[66] Ramzan A, Hafiz I, Ahmad T, Abbasi N (2010). Effect of priming with potassium nitrate and dehusking on seed germination of gladiolus (*Gladiolus alatus*). Pak. J. Bot..

[67] Yücel E, Yilmaz G (2009). Effects of different alkaline metal salts (NaCl, KNO_3_), acid concentrations (H_2_SO_4_) and growth regulator (GA_3_) on the germination of *Salvia cyanescens* Boiss. & Bal. seeds. Gazi Univ. J. Sci..

[68] Abnavi MS, Ghobadi M (2012). The effects of source of priming and post-priming storage duration on seed germination and seedling growth characteristics in wheat (*Triticum aestivem* L.). J. Agric. Sci..

[69] Hartmann HT, Kester DE (1975). Plant Propagation: Principles and Practices.

[70] Baiyeri K, Mbah B (2006). Effects of soilless and soil-based nursery media on seedling emergence, growth and response to water stress of African breadfruit (*Treculia africana* Decne). Afr. J. Biotech..

[71] Ali AS, Elozeiri AA (2017). Metabolic processes during seed germination. Adv. Seed Biol..

[72] Ameen Al-Imam, N. & Saleh Al-Jubury, Y. *V International Symposium on Pistachios and Almonds*, 245–252 (2009).

[73] Gupta SD, Agarwal A (2017). Light Emitting Diodes for Agriculture.

[74] Solano CJ, Hernández JA, Suardíaz J, Barba-Espín G (2020). Impacts of LEDs in the red spectrum on the germination, early seedling growth and antioxidant metabolism of pea (*Pisum sativum* L.) and melon (*Cucumis melo* L.). Agriculture.

[75] Pierik R, de Wit M (2014). Shade avoidance: Phytochrome signalling and other aboveground neighbour detection cues. J. Exp. Bot..

[76] Zhang T, Maruhnich SA, Folta KM (2011). Green light induces shade avoidance symptoms. Plant Physiol..

[77] Folta KM, Maruhnich SA (2007). Green light: a signal to slow down or stop. J. Exp. Bot..

[78] Kim, H. H., Wheeler, R. M., Sager, J. C., Gains, G. & Naikane, J. *V International Symposium on Artificial Lighting in Horticulture* 711, 111–120 (2005).

[79] Ichimura, M., Watanabe, H., Amaki, W. & Yamazaki, N. *VI International Symposium on Light in Horticulture* 907, 91–94 (2009).

[80] Carvalho SD, Schwieterman ML, Abrahan CE, Colquhoun TA, Folta KM (2016). Light quality dependent changes in morphology, antioxidant capacity, and volatile production in sweet basil (*Ocimum basilicum*). Front. Plant Sci..

[81] Sipos L (2021). Optimization of basil (*Ocimum basilicum* L.) production in LED light environments: A review. Sci. Horticult..

[82] Poorter H, Remkes C (1990). Leaf area ratio and net assimilation rate of 24 wild species differing in relative growth rate. Oecologia.

[83] Hampton, J. G. & TeKrony, D. M. (International Seed Testing Association Zurich, 1995).

