# Supplementary material for: Promotion of seed germination and early plant growth by KNO3 and light spectra in Ocimum tenuiflorum using a plant factory
Source: Sci Rep. 2022 Apr 29;12:6995. doi: 10.1038/s41598-022-11001-5 (PMC9054764; doi:10.1038/s41598-022-11001-5)
Supplement: Supplementary file 1 — Supplementary Information. [file 41598_2022_11001_MOESM1_ESM.pdf]

Supplementary document S1:

Promotion of seed germination and early plant growth by KNO_3 and light spectra in *Ocimum tenuiflorum* using a plant factory

Akira Thongtip[1], Kriengkrai Mosaleeyanon[1], Siripar Korinsak[1], Theerayut Toojinda[1], Clive Terence Darwell[1], Preuk Chutimanukul[2] and Panita Chutimanukul[1,*]

*****Corresponding author**:** Panita Chutimanukul, Tel. +6625646700, +66841636459

Email**:** panita.chu@biotec.or.th, p.chutimanukul@gmail.com

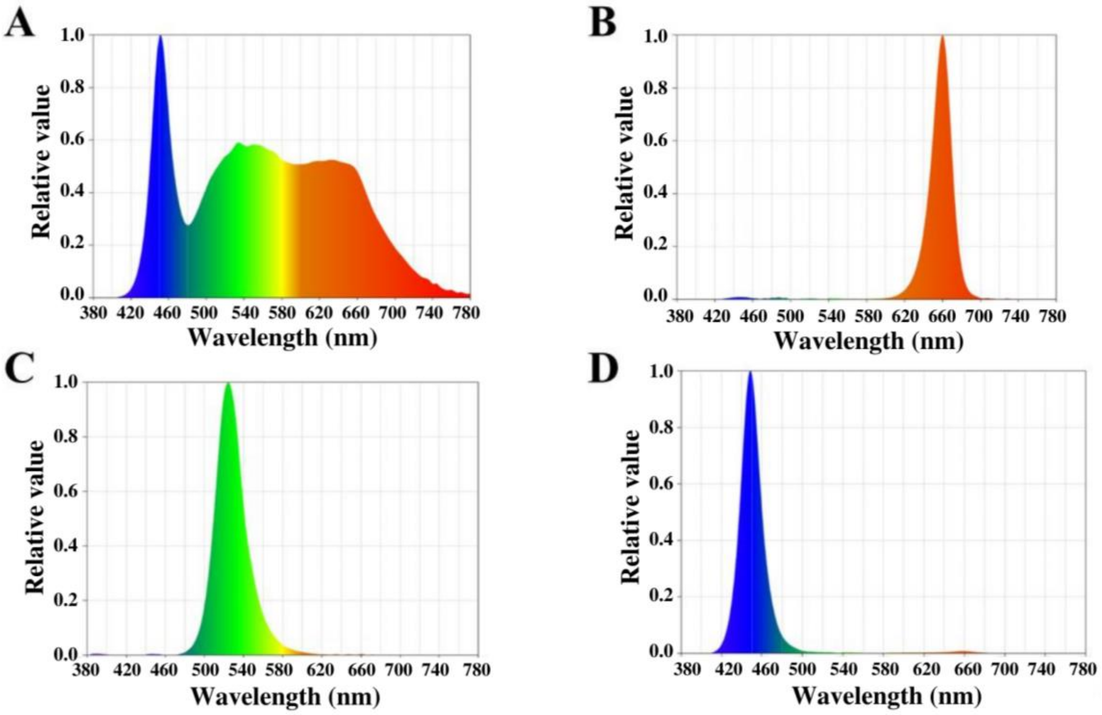

Figure S1. The relative spectra of the light treatment consist of white LEDs (λ = 740 nm) (A), monochromatic red (λ = 740 nm) (B), green (λ = 523 nm) (C) and blue (λ = 448 nm) (D) at 150 $\mu mol\ m^{-2}\ s^{-1}$.

Supplementary document S2:

Promotion of seed germination and early plant growth by KNO_3 and light spectra in *Ocimum tenuiflorum* using a plant factory

Akira Thongtip[1], Kriengkrai Mosaleeyanon[1], Siripar Korinsak[1], Theerayut Toojinda[1], Clive Terence Darwell[1], Preuk Chutimanukul[2] and Panita Chutimanukul[1,*]

*Corresponding author: Panita Chutimanukul, Tel. +6625646700, +66841636459

Email: panita.chu@biotec.or.th, p.chutimanukul@gmail.com

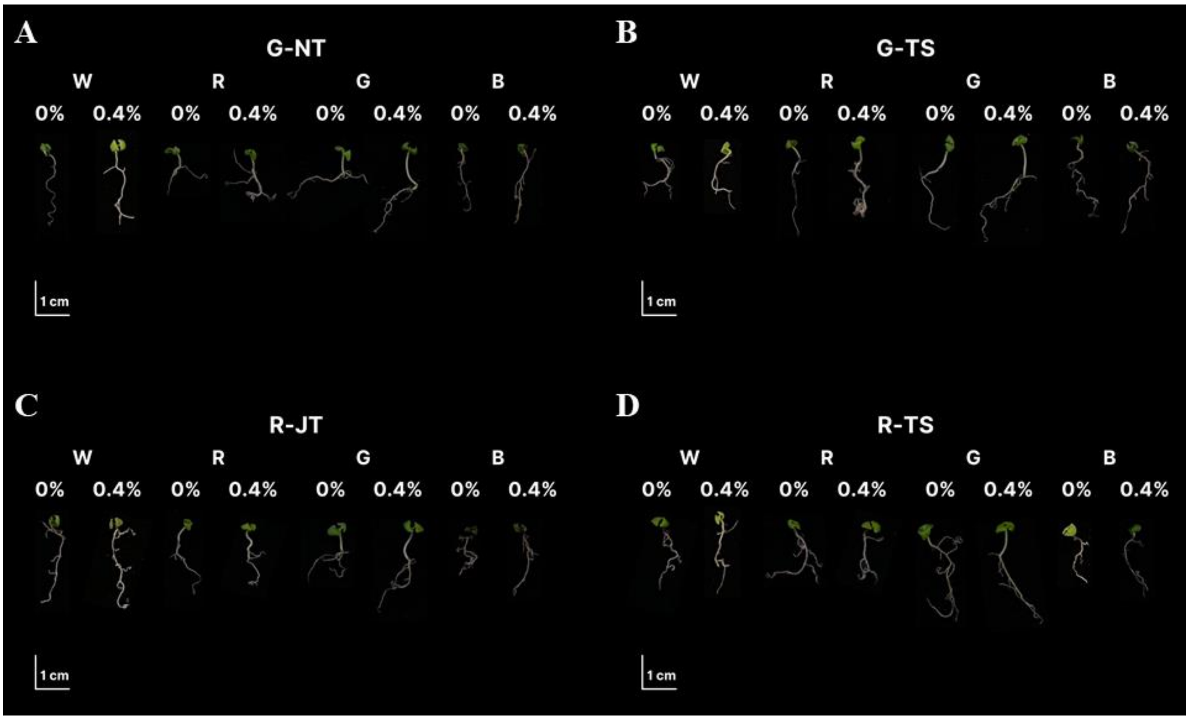

Figure S2. The phenotypes of holy basil seedlings from 4 varieties; G-NT (A), G-TS (B), R-JT (C) and R-TS (D) on four light treatments; white LEDs (W), monochromatic red (R), monochromatic green (G) and monochromatic blue (B) with 0% and 4% of KNO_3 priming before transplanting at 15 days after sowing in PFAL system.

Supplementary Table S1:

Promotion of seed germination and early plant growth by KNO_3 and light spectra in *Ocimum tenuiflorum* using a plant factory

Akira Thongtip[1], Kriengkrai Mosaleeyanon[1], Siripar Korinsak[1], Theerayut Toojinda[1], Clive Terence Darwell[1], Preuk Chutimanukul[2] and Panita Chutimanukul[1,*]

*Corresponding author: Panita Chutimanukul, Tel. +6625646700, +66841636459

Email: panita.chu@biotec.or.th, p.chutimanukul@gmail.com

Table S1. Root fresh and dry weight of early plant growth from four light treatments; white LEDs (W), monochromic red (R), green (G) and blue (B) LEDs with 0% and 4% of KNO_3 priming among four holy basil varieties; G-NT (A), G-TS (B), R-JT (C) and R-TS (D) subsequently grown with white LEDs for 15 days under fully controlled environments.

KNO_3 concentration	Light treatment	Root fresh weight (mg)			
		G-NT	G-TS	R-JT	R-TS
0%	W	66.8±4.81	49.1±3.63c	21.7±0.97b	50.7±3.41b
	R	55.4±0.86	79.1±0.83b	55.2±1.87a	22.7±1.47c
	G	52.1±4.76	151.1±5.55a	63.3±6.01a	78.9±1.47a
	B	52.3±2.99	68.0±2.81b	65.0±10.63a	78.8±3.51a
0.4%	W	46.2±18.17b	50.0±1.92b	48.3±2.17b	55.4±5.42b
	R	28.3±2.03b	50.2±5.48b	69.6±8.95a	42.7±2.78b
	G	82.4±7.05a	222.4±18.78a	68.8±6.59a	76.6±3.33a
	B	34.4±2.25b	53.4±2.71b	65.1±8.76a	21.2±3.16c

KNO_3 concentration	Light spectrum	Root dry weight (mg)			
		G-NT	G-TS	R-JT	R-TS
0%	W	4.2±0.48b	10.2±1.89b	2.3±0.51c	4.1±0.48
	R	6.7±0.55a	2.2±0.28c	5.6±1.20bc	4.7±2.65
	G	8.1±0.51a	22.4±2.68a	11.2±1.32a	4.4±1.47
	B	3.8±0.62b	4.3±0.36c	14.1±3.33a	7.3±3.58
0.4%	W	2.7±0.39b	3.9±0.56b	6.9±3.05	5.9±2.66
	R	2.5±0.58b	6.2±1.38b	9.1±2.73	11.1±2.94
	G	20.5±1.47a	46.5±9.98a	16.2±7.95	4.7±0.24
	B	4.4±0.65b	7.9±1.73b	21.8±3.65	1.4±0.12

Data are mean values ± SE (n = 4) with four fifty seeds in a replication. Different letters indicate significant difference between lines at $p < 0.05$.
